# Supplementary figures and images for: TLR3 and TLR7 RNA Sensor Activation during SARS-CoV-2 Infection
Source: Microorganisms. 2021 Aug 26;9(9):1820. doi: 10.3390/microorganisms9091820 (PMC8465566; doi:10.3390/microorganisms9091820)

Supplementary Figure S1

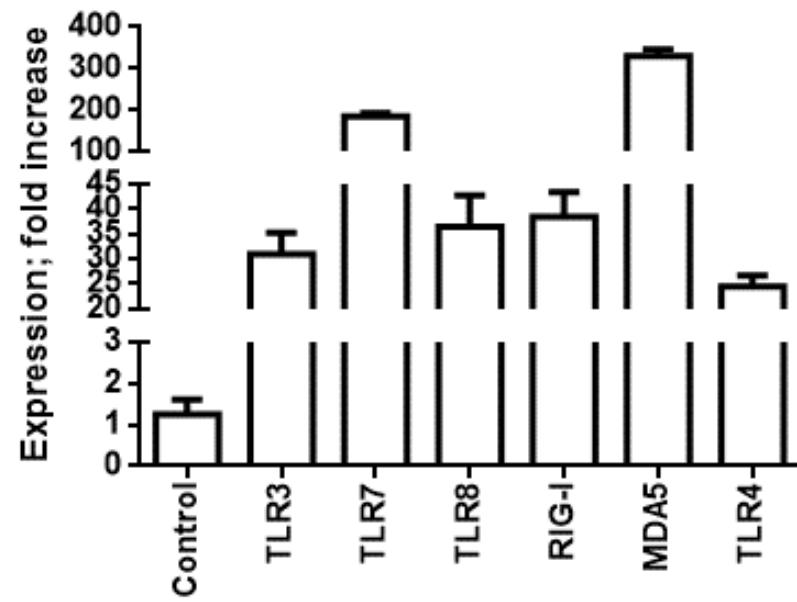

Supplementary Figure S2

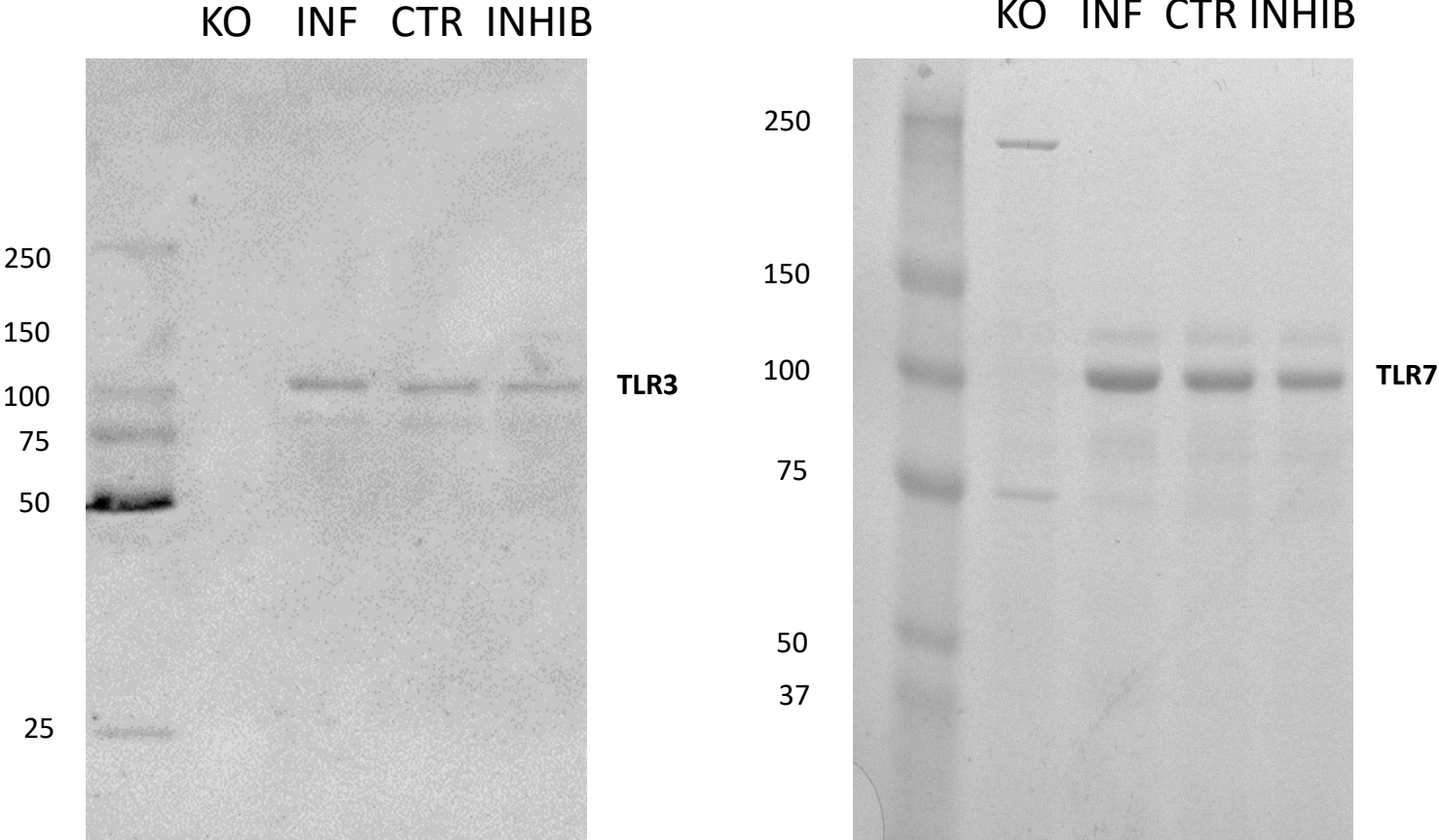

Supplementary Figure S3

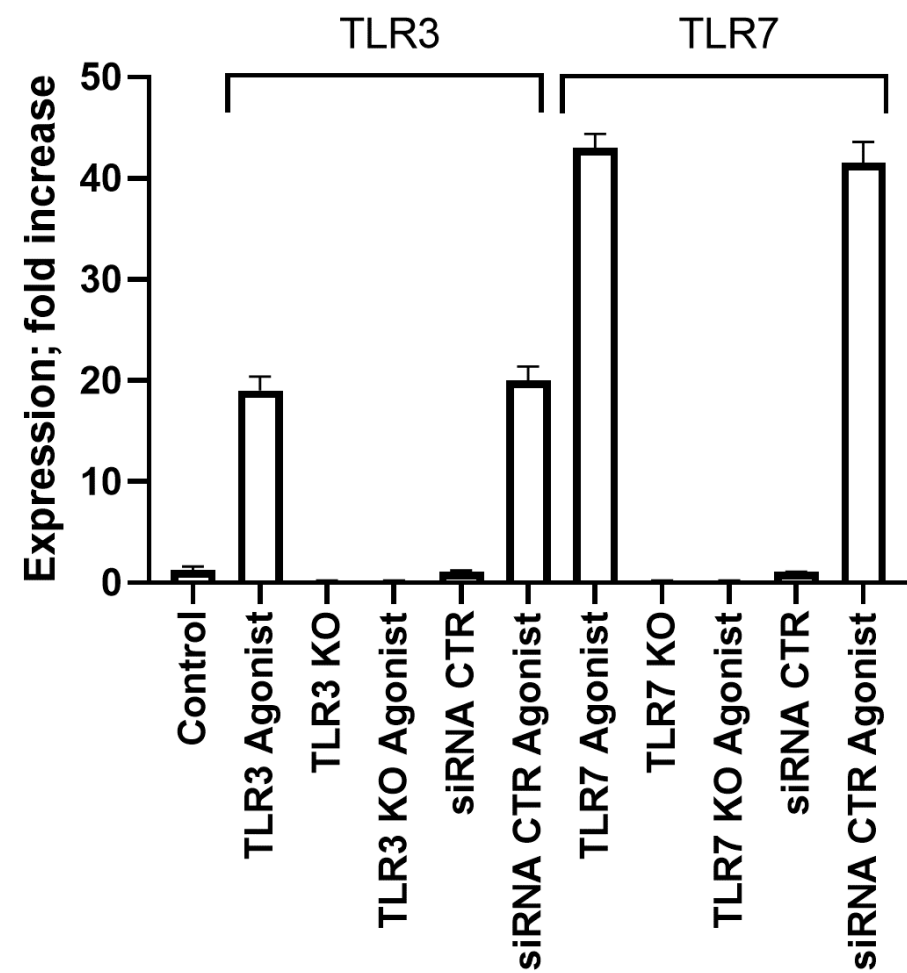

Supplement: Supplementary file 1 [file microorganisms-09-01820-s001.zip › microorganisms-1332308-supplementary.pdf]
